# Long-Term Follow-Up of the Quality of Life of Endometriosis Patients after Surgery: A Comparative Study

**DOI:** 10.3390/jcm13185641

**Published:** 2024-09-23

**Authors:** Alice Wenzl, Rene Wenzl, Manuela Gstoettner, Lorenz Kuessel, Heinrich Husslein, Jana Heine, Lejla Sandrieser, Christine Bekos, Alexandra Perricos-Hess

**Affiliations:** 1 Department of Obstetrics and Gynecology, Spitalspartner Ordensklinikum Linz und Konventhospital Barmherzige Brueder, Seilerstaette 2, 4020 Linz, Austria; 2 Department of Obstetrics and Gynecology, Medical University of Vienna, Waehringer Guertel 18-20, 1090 Vienna, Austria; rene.wenzl@meduniwien.ac.at (R.W.); manuela.gstoettner@meduniwien.ac.at (M.G.); lorenz.kuessel@meduniwien.ac.at (L.K.); heinrich.husslein@meduniwien.ac.at (H.H.); jana.heine@meduniwien.ac.at (J.H.); lejla.sandrieser@meduniwien.ac.at (L.S.); christine.bekos@meduniwien.ac.at (C.B.); alexandra.perricos-hess@meduniwien.ac.at (A.P.-H.)

**Keywords:** endometriosis, quality of life, long-term follow-up, EHP-30, surgery

## Abstract

**Background/Objectives:** This study investigated the long-term effects of the surgical removal of endometriotic lesions on quality of life in endometriosis patients. A sub-analysis explored different subtypes of endometriosis, hormonal influence, and the need for reoperation. **Methods:** The study was conducted at the Certified Endometriosis Center of the Medical University of Vienna. Included in the study were patients who had undergone the complete surgical removal of endometriotic lesions between 2014 and 2018. Patients were asked to complete the Endometriosis Health Profile-30 preoperatively, at the short-term follow-up (six to ten weeks postoperatively), and at the long-term follow-up (median of 48 months postoperatively). **Results:** A total of 87 patients completed the Endometriosis Health Profile-30 at the three time points. At the long-term follow-up, the reoperation rate was 8.0%. Significant improvements in the overall quality of life (*p* < 0.001; median decrease from 45.0 to 11.7) and in the categories of “pain”, “control and powerlessness”, “social support”, “emotional well-being”, and “self-image” at the long-term follow-up compared to preoperative values were observed (*p*-values < 0.01). The sub-analysis showed that patients with deep-infiltrating endometriosis (*p* < 0.001; median decrease from 49.2 to 10.0) and adenomyosis (*p* < 0.02; median decrease from 37.5 to 0.0) had the most pronounced long-term postsurgical benefits in terms of quality of life. Patients with (*p* < 0.001; median decrease from 45.0 to 1.7) and without (*p* < 0.001; median decrease from 45.42 to 12.5) hormonal influence showed significant improvements in overall quality of life compared to preoperative values. Patients without reoperation demonstrated improved long-term quality of life compared to the preoperative (*p* < 0.001; median decrease from 45.8 to 9.6) and short-term follow-up results (*p* < 0.005; median decrease from 19.2 to 9.6). Participants who underwent reoperation showed no improvement in quality of life at the long-term follow-up. **Conclusions**: The surgical removal of endometriotic lesions has a positive long-term impact on the quality of life, as measured by Endometriosis Health Profile-30.

## 1. Introduction

Endometriosis, one of the most common gynecological disorders, is a benign, estrogen-dependent, chronic inflammatory disease [1,2] that affects approximately ten percent of women of reproductive age [2]. 

Due to the inflammatory response induced by ectopic endometrial tissue and, consecutively, the deformation of pelvic organs, these patients can suffer from a variety of symptoms. The range of the severity of these symptoms is very wide, from minimal to heavy and subjectively intolerable pain symptoms. Typical symptoms associated with endometriosis include dysmenorrhea, dyspareunia, dyschezia, dysuria, and non-menstrual pelvic pain [3]. Endometriosis-related pain symptoms can negatively influence a woman’s daily life routine and can consequently affect the patient’s quality of life [4].

The reduced quality of life concerns different areas. Patients complain about limitations in their personal, as well as their work, lives [5]. Mental well-being, as well as social relationships, can be negatively affected in patients with endometriosis [6]. 

Increased stress levels and a tendency to suffer from depression and anxiety are reported by these patients. Regarding their work lives, affected women complain about a disturbed ability to work and many days of work leave because of endometriosis-related pain [5]. 

Furthermore, sexual function may be adversely affected [7]. 

Many studies have shown that endometriosis is often associated with infertility. Up to fifty percent of infertile women are diagnosed with endometriosis [5,8].

Endometriosis is a chronic disease, and therefore, long-term therapy is necessary. Clinical symptoms, in combination with the localization and extent of endometriotic lesions, influence the choice of appropriate treatment. The goal is to select the least invasive but most effective treatment option [8]. For patients with pelvic pain and no desire for immediate pregnancy, medical treatment should be offered as a first-line therapy [9]. Surgical treatment options should be carefully and individually evaluated if medical therapy is inefficient. However, surgery is indicated in cases of associated severe stenosis of the urinary and/or gastrointestinal tract, as well as in cases of ovarian masses with suspected malignancy [8,10]. 

The goal of surgery is to resect all ectopic lesions that can be visually verified and to reestablish the anatomy of the pelvic organs [8]. 

Surgical therapy for endometriosis is associated with special risks for complications, such as the reduction of the ovarian reserve, the development of adhesions, and common surgical risks, such as wound infection, thromboembolic events, and the injury of organs [11]. 

The adjuvant use of hormones after surgery potentially increases the time until the recurrence of endometriosis-related pain symptoms [8]. 

Many studies have proven that complete surgical removal of endometriotic lesions leads to a positive impact on quality of life (QoL), including sexual QoL [12,13,14,15]. 

When comparing the surgical removal of endometriotic lesions with placebo surgery (in the form of purely diagnostic laparoscopy), the prospective, randomized study by Abbott et al. demonstrated significant pain reduction six months after surgery only in the group that had undergone the removal of ectopic lesions [16,17]. 

Nowadays, endometriosis surgery is usually performed via minimal invasive access, i.e., a laparoscopic approach, instead of open surgery via laparotomy. Laparoscopic surgery is associated with a significantly lower risk of complications, a shorter recovery time, lower costs, and a decreased time of hospital stay compared to open surgery [18]. Furthermore, laparoscopy offers the surgeon an overview of the whole abdominal cavity, including the pelvic area, and can therefore potentially exclude other relevant pathologies [19]. In addition to conventional laparoscopy, robot-assisted laparoscopy is also applicable. No significant advantages, such as better effect on pain symptoms, reduced blood loss, or other lower complication rates, were perioperatively found for robotic-assisted laparoscopy compared to conventional laparoscopy [20].

Very limited data are available on the long-term impact of the quality of life after the surgical removal of endometriotic lesions. This fact demonstrates the importance of further studies in this field [21]. 

The aim of this study is to investigate the long-term effects of the surgical removal of endometriotic lesions on quality of life. A sub-analysis explores different subtypes of endometriosis, hormonal influence, and the need for reoperation. 

As no correlation between the extent of the disease and the severity of symptoms has been detected, it is important to take the subjective perceptions of restrictions on the quality of life of endometriosis patients into account [22]. The Endometriosis Health Profile-30 (EHP-30, as shown in the Supplementary Materials) is the most comprehensive health-related quality of life questionnaire that was specifically developed and used only for patients with endometriosis. This questionnaire is more sensitive to typical symptoms caused by this disease. Additionally, this scale is able to illustrate changes in the health status of patients with endometriosis [23]. 

## 2. Materials and Methods

### 2.1. Study Design

This prospective longitudinal cohort study using questionnaires was conducted at the Certified Endometriosis Center of the Medical University of Vienna certified by the EuroEndoCert. This study was approved by the Ethics Committee of the Medical University of Vienna (EK no. 1907/2019). Prior to the voluntary participation, written informed consent was obtained. Additionally, all patient data, including the consent form, was stored at the Certified Endometriosis Center of the Medical University of Vienna. 

We compared our long-term follow-up data with preoperative and short-term follow-up (six to ten weeks postoperative) data. 

### 2.2. Patients

Our study is a long-term follow-up of the previously published study by Tiringer et al., in which 114 patients were recruited [24]. The previous study evaluated the postoperative change in QoL six to ten weeks after surgery, measured by using the EHP-30, of patients who had undergone the complete surgical removal of endometriotic lesions at the certified Endometriosis Center of the Department of Obstetrics and Gynecology at the Medical University of Vienna between 2014 and 2018. 

In our present study, the same patients completed the EHP-30 at a median follow-up of 48 months to evaluate the long-term improvement in QoL after surgery. The patients also received a patient history questionnaire created by the Endometriosis Center of the Medical University of Vienna, to collect anamnesis, clinical, and pregnancy data on the study participants in the long-term follow-up. The patients received the patient history questionnaire simultaneously with the EHP-30. Anamnesis items that were captured were age, BMI, menarche, smoking habits, and partnership. 

The collected clinical parameters were time of follow-up in months, duration of bleeding, hormonal therapy, type of hormonal therapy, overall pain score, pain days per month, dysmenorrhea, dyspareunia, sex life, dysuria, abdominal symptoms, and whether the patient had undergone hysterectomy. 

The pregnancy data included a desire to have children, pregnancies since surgery, and breastfeeding at the time of follow-up. 

Additionally, we assessed the reoperation rate, as well as a general patient history form, focusing on hormonal influence, including intake of hormonal therapy, pregnancy, or breastfeeding. Based on the histological findings obtained during surgery, we investigated the different subtypes of endometriosis. 

We excluded patients with malignant diseases, chronic infectious diseases (such as HIV, hepatitis B/C, tuberculosis), or systemic autoimmune diseases acquired during the follow-up period from this study. 

### 2.3. Endometriosis Health Profile-30

The Endometriosis Health Profile-30 (EHP-30) was specifically developed and used only for patients with endometriosis in order to evaluate QoL. Based on the results of interviews with affected women who presented with pain symptoms and described the impact of endometriosis on their QoL, this validated and reliable questionnaire was successfully established [23]. 

The questionnaire is divided into two parts; the first comprises 30 core questions, and the second modular part consists of 23 questions and applies only to some of the affected women [25]. 

A great strength of EHP-30 is the easy wording of the questions, whereby the patients are able to fill in the questionnaire without a doctor’s guidance. The downside is the large quantity of questions. Consequently, the response time takes approximately ten to fifteen minutes, which can result in lower compliance [23].

The availability and validation of the questionnaire in some languages beyond English allows international access and use [23].

Very limited data are available on the long-term impact of surgical removal of endometriotic lesions on QoL [21,26]. Thus, the aim of our study was to evaluate the long-term effect of surgery on the QoL of patients with endometriosis. In addition, a sub-analysis was performed comparing the outcome between the different subtypes of endometriosis (peritoneal, endometrioma, ovarian endometriosis [OMA], deep-infiltrating endometriosis [DIE], DIE + OMA, adenomyosis), as well as to compare the QoL of patients under hormonal influence (hormonal treatment, pregnancy or breastfeeding) to patients without hormonal influence, and finally to compare the QoL after a potential reoperation during the follow-up period in patients with no reoperation.

### 2.4. Statistical Analysis

Patients were divided into five groups based on the endometriosis subtypes that were surgically diagnosed (peritoneal endometriosis, OMA, DIE, DIE + OMA, and adenomyosis). 

To analyze the results of the EHP-30, the subscales were converted as described by Jones et al. [25]. The five QoL domains (pain, control and powerlessness, social support, emotional well-being, and self-image) include 30 questions with five different response options (never, seldom, rarely, often, always). Each response option was assigned to point values within the range of zero to four. The lower the point value, the lower the frequency of the health problems that occurred; the higher the point value, the higher the frequency of the health issues that occurred (0 = never, 1 = seldom, 2 = rarely, 3 = often, 4 = always). Afterward, the 30 questions were combined into five different subscales based on the QoL domain (pain, control and powerlessness, social support, emotional well-being, and self-image). For example, the questions concerning pain were all included in the pain scale and equally proceeded in each subscale. The points of one scale were summed up, divided by the total possible score, and multiplied by 100. Consequently, scores between 0 and 100 were possible (0 = no restriction in the quality of life, 100 = maximum restriction in the quality of life). Each subscale was equally calculated [25]. 

The study participants (*n* = 87) completed the main part of the EHP-30 at the following three time points: preoperative, at the short-term follow-up (six to ten weeks after surgery), and at the long-term follow-up (median of 48 months after surgery). The medians with interquartile ranges were given between the range of 0 to 100; the lower they were, the less influence, and the higher they were, the more influence in the quality of life in patients with endometriosis. 

The scores of the main part of the EHP-30 were tested for normality using the Shapiro–Wilk test. If not normally distributed, the scores were statistically analyzed with the Wilcoxon signed rank test, which was used for paired samples to compare the medians of the scores between preoperative and long-term follow-up and between short-term follow-up (six to ten weeks postoperative) and long-term follow-up (median of 48 months postoperative) of the whole study population regarding the main domains of QoL based on the EHP-30.

*p*-values < 0.05 were considered statistically significant.

Finally, the postoperative EHP-30 score was multivariately analyzed via ordinal logistic regression. *p*-values < 0.05 were considered statistically significant. Additionally, the change from baseline was plotted as a boxplot, with dots representing each patient and lines showing the actual change (with colors representing the magnitude of the change). 

## 3. Results

### 3.1. Recruitment and Demographics

All of the 114 women who took part in the study by Tiringer et al. in 2022 [24] were contacted by our study team either via email or phone, and the questionnaires were sent by post. After three unsuccessful attempts to reach the patient, we tried to reach the designated contact. A total of 88 patients filled out and returned the questionnaires, corresponding to a response rate of 77.2%. 

A total of 87 patients were finally included in this study. One patient was excluded because of breast cancer that was diagnosed during the follow-up period. The majority of study participants, eighty patients, filled out the questionnaires at home and sent them back via prepaid envelopes. Only seven women took the opportunity to arrange an appointment at our clinic to complete the EHP-30 and patient history questionnaire in the hospital. 

If the endometriosis patients still suffered and complained of pain symptoms, they were offered a doctor’s appointment at our clinic. Two patients took the opportunity for a doctor’s appointment.

Table 1 shows the patient characteristics at the long-term follow-up. The median long-term follow-up period was 48 months (interquartile range, 39 to 56 months). The median age of the study participants was 36 years (interquartile range, 31 to 42 years). Seven out of eighty-seven patients (8.0%) underwent another endometriosis surgery during the time of follow-up. Sixteen patients (18.4%) were under hormone therapy at the long-term follow-up. Nine patients (10.3%) had undergone hysterectomy. 

As visualized in Table 2, 31 patients, the majority of the study participants, had deep-infiltrating endometriosis without ovarian involvement. Nineteen patients had ovarian endometriosis. The peritoneal subtype was presented in fifteen patients, closely followed by the deep-infiltrating subtype, including ovarian endometriosis, in fourteen patients. Only 8 of the 87 study participants had adenomyosis. 

### 3.2. Impact of Patient Characteristics on EHP-30

Our multivariate analysis found that neither age, smoking, nor hormone intake influenced the EHP-30 score at the time of long-term follow-up. However, the predictor pregnancy during the follow-up period had a significant positive impact on our primary outcome parameter (postoperative EHP-30 score), as presented in Table 3. 

**Figure 1:**
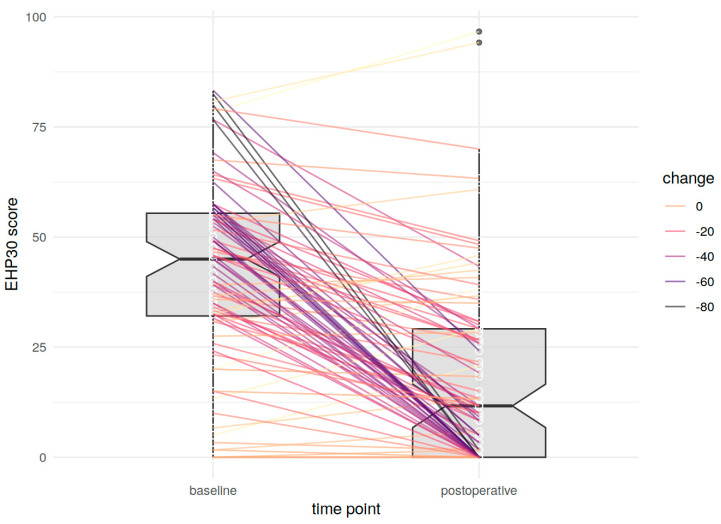
Changes in EHP-30 scores by each patient from preoperative to long-term follow-up.

The changes by each patient are presented in Figure 1 by boxplots.

### 3.3. Results of EHP-30

**Figure 2:**
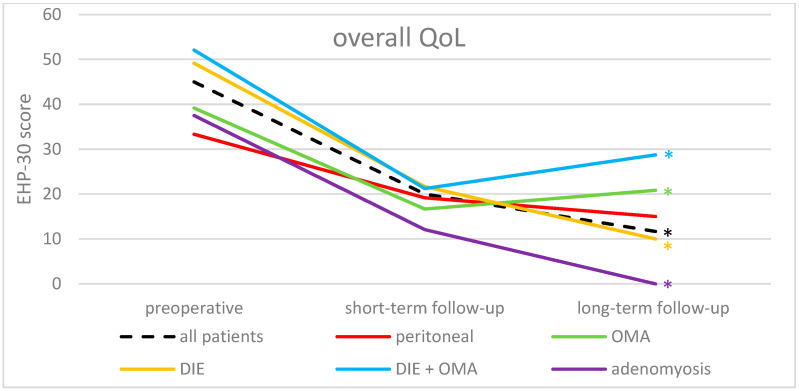
Results of EHP-30 overall QoL of the whole study population and divided by the endometriosis subtypes at the three time points. * = significant improvement between preoperative and long-term follow-up (*p*-value < 0.05). QoL, quality of life; OMA, endometrioma; DIE, deep-infiltrating endometriosis.

The overall QoL of all study participants was significantly improved comparing long-term follow-up with preoperative EHP-30 scores (*p* < 0.001; median decrease from 45.0 to 11.7), as well as with short-term follow-up scores (*p* = 0.019; median decrease from 20.0 to 11.7) (Figure 2). Significant improvements were also demonstrated in all five endometriosis subtypes (peritoneal (*p* < 0.03; median decrease from 33.3 to 15.0), OMA (*p* < 0.003; median decrease from 39.2 to 20.8), DIE (*p* < 0.001; median decrease from 49.2 to 10.0), DIE + OMA (*p* < 0.04; median decrease from 52.1 to 28.8), and adenomyosis (*p* < 0.02; median decrease from 37.5 to 0.0), comparing preoperative and long-term follow-up results.

Furthermore, the subtypes DIE (*p* < 0.05; median decrease from 21.7 to 10.0) and adenomyosis (*p* < 0.03; median decrease from 12.1 to 0.0) even showed improvements in the overall QoL comparing short-term follow-up and long-term follow-up time points.

**Figure 3:**
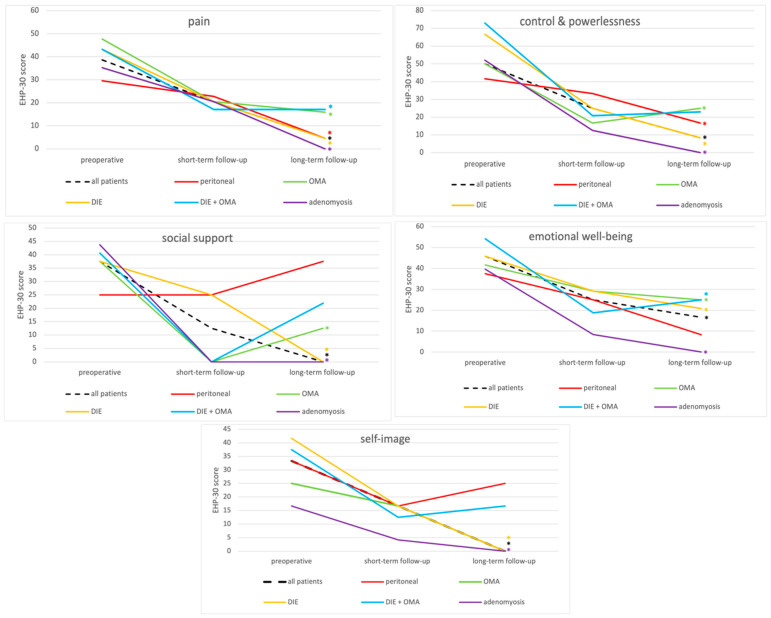
Results of EHP-30 subscales (pain, control and powerlessness, social support, emotional well-being, self-image) of the whole study population (*n* = 87) and divided by the endometriosis subtypes at the three time points. * = significant improvement between preoperative and long-term follow-up (*p*-value < 0.05). QoL, quality of life; OMA, endometrioma; DIE, deep-infiltrating endometriosis.

The five subscales of EHP-30 demonstrated significant improvements between preoperative and long-term follow-up results (*p* < 0.001), of which “pain” (*p* < 0.005; median decrease from 20.5 to 4.6) and “control and powerlessness” (*p* < 0.05; median decrease from 25.0 to 8.3) also showed significant improvements between short-term follow-up and long-term follow-up in all study participants (Figure 3).

The subgroups of DIE (*p* < 0.001) or adenomyosis (*p* < 0.033) demonstrated significant improvements in all EHP-30 scales comparing preoperative with long-term follow-up results. 

In addition, the EHP-30 subscale “pain” demonstrated an improvement in all endometriosis subtypes at the long-term follow-up compared to preoperative results (*p* < 0.038) (Figure 3).

**Figure 4:**
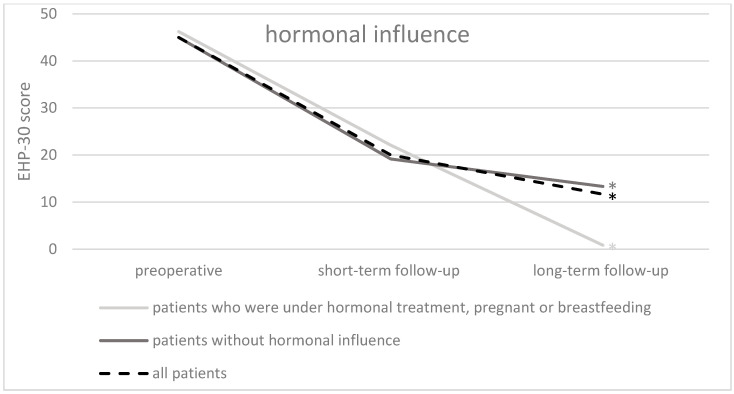
Results of EHP-30 overall QoL of the whole study population (*n* = 87) and divided by hormonal influence and without hormonal influence at the three time points. * = significant improvement between preoperative and long-term follow-up (*p*-value < 0.05).

Comparing preoperative and long-term follow-up overall QoL, patients under hormonal influence (intake of hormonal therapy, pregnancy, or breastfeeding) (*p* < 0.001; median decrease from 45.0 to 1.7), as well as patients without hormonal influence (*p* < 0.001; median decrease from 45.42 to 12.5) demonstrated significant improvements (Figure 4).

**Figure 5:**
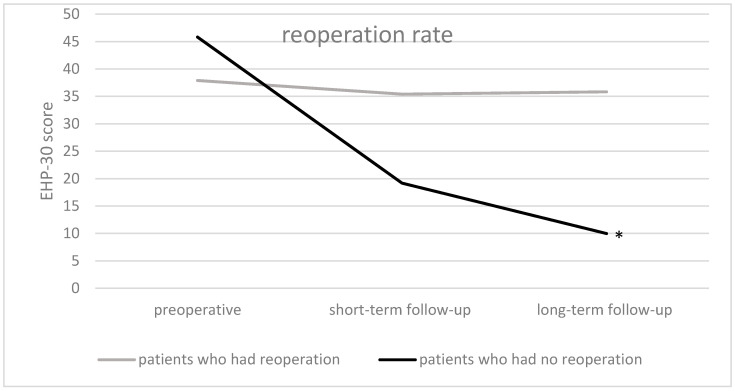
Results of EHP-30 overall QoL of the whole study population (*n* = 87) by need of reoperation at the three time points. * = significant improvement between preoperative and long-term follow-up (*p*-value < 0.05).

The reoperation rate due to endometriosis recurrence at the long-term follow-up was 8.0% (*n* = 7). Patients who had no reoperation showed significant overall QoL improvements comparing preoperative and long-term follow-up results (*p* < 0.001; median decrease from 45.8 to 9.6), as well as comparing short-term and long-term follow-up (*p* < 0.005; median decrease from 19.2 to 9.6) (Figure 5). 

Patients who underwent a reoperation did not show improvements in the overall QoL comparing preoperative and long-term follow-up results (Figure 5).

## 4. Discussion

Endometriosis is a chronic inflammatory disease that can dramatically negatively influence the QoL of affected patients. The EHP-30 serves as a validated measurement tool addressing the QoL of patients with endometriosis. The aim of our study was to compare the QoL at a long-term follow-up with preoperative scores. Our study is a long-term follow-up of the previously published study by Tiringer et al., in which 114 patients were recruited [24]. The previous study evaluated the postoperative change in QoL six to ten weeks after surgery, which was measured by using the EHP-30. 

We made a strong effort to reach every patient of the 114 patients who were included in the study by Tiringer et al. We contacted them by phone and mail, and after three unsuccessful attempts, we even tried to reach the contact person. We are aware that a response rate of 77.2% is a potential bias and can impact the study results.

The systematic review by Jones et al. found that the EHP questionnaires, particularly the EHP-30 and EHP-5, are effective and widely used tools for measuring the QOL outcomes in women with endometriosis in the last two decades. The review highlighted that these profiles reliably capture a broad range of physical, emotional, and social impacts of endometriosis, making them valuable for both clinical practice and research. The EHP-30 however has limitations, such as a narrow focus and difficulty comparing with other tools. Relying only on the EHP-30 might miss important aspects of quality of life, so using additional measures could provide a detailed assessment [27]. 

Our study results demonstrate that the surgical removal of endometriotic lesions significantly improves QoL in the long run at a median time follow-up of 48 months. 

Regarding the subtypes of endometriosis, patients with DIE and adenomyosis showed the most pronounced postsurgical effect on quality of life comparing preoperative and long-term follow-up. The reoperation rate was 8.0% during long-term follow-up, comprising all subtypes of endometriosis.

Several studies, including Dogan et al., Jones et al., and Rindos et al., have reported positive QoL short-term outcomes after the surgical removal of endometriotic lesions based on the EHP-30. Dogan et al. [28] demonstrated significant improvements three to six months after surgery regarding “pain”, “control and powerlessness”, “emotional well-being”, “social support”, “sexual intercourse”, and “concerns about medical profession”. Jones et al. showed QoL changes four months after surgery based on the EHP-30 in all domains except for “social support”, “concerns about treatment”, and “feelings about fertility” [29]. 

The study by Rindos et al., with 46 patients, showed significant improvements in all five main scales of the EHP-30 four weeks after surgery. These improvements persisted at a long-term follow-up of 2.6 to 6.8 years [21], which supports our long-term follow-up results. 

Abbott et al., using other QoL measurement tools not specifically developed for patients with endometriosis, such as EQ-5D and Short-Form 12, demonstrated significant QoL improvements (pain and sexual function) two to five years after surgery [30]. 

In addition, our results in the overall QoL and the categories “pain” and “control and powerlessness” showed statistically significant improvements comparing long- with short-term follow-up. However, these results were not reported in a similar study by Rindos et al. [21]. 

The study by Turco et al., which included 50 patients affected by DIE who underwent segmental colorectal resection, presented significant improvements at a follow-up period of 42.5 months in all scales of the main and modular parts of the EHP-30, except for “feelings about fertility” [31].

In the literature, only a few studies have described the surgical effect on QoL, sub-analyzed by the endometriosis subtype. Many studies, however, have only evaluated the long-term outcome of QoL in patients with DIE. A strength of our study is the sub-analysis of endometriosis subtypes. Patients with DIE and adenomyosis demonstrated significant improvements in all five QoL categories compared to preoperative and long-term follow-up.

Patients with DIE and adenomyosis exhibited significant improvements across all five QoL categories comparing preoperative and long-term follow-up. The study by Turco et al. supported our findings, which included 50 patients affected by DIE who underwent segmental colorectal resection, with significant improvements at a follow-up period of 42.5 months in all scales of the main part of the EHP-30 [31]. Benbara et al. also supported our findings, reporting positive long-term QoL outcomes in most categories after 42 months based on the EHP-30 of patients with DIE after colorectal resection [32]. 

Concerning the adenomyosis subgroup, QoL improvements were most pronounced, potentially influenced by a higher rate of hysterectomy.

Moreover, our study delves into the influence of hormonal treatments in the long-term follow-up, revealing significant QoL improvements in both groups, those under hormonal influence and those without hormonal influence. 

Alkatout et al.’s study demonstrated the efficacy of adjuvant hormonal treatment for the treatment of endometriosis-associated symptoms compared to surgery or hormonal treatment alone at a follow-up time of up to six months [33]. 

Capezzuoli et al. showed that hormonal treatment before and after surgery had a positive effect on pain symptoms (especially dysmenorrhea) and a lower rate of reoperations [34]. Thus, the combination of surgery and hormonal treatment appears to be effective for long-term QoL outcomes in patients with endometriosis. 

The study by Biasioli et al. suggests that hormone therapy alone may not fully enhance quality of life or sexual function, and a multimodal, multidisciplinary approach may be more effective [35]. 

Initially, it was assumed that pregnancy can improve endometriosis-related symptoms due to hormonal changes; however, this fact is only supported by a limited number of studies. Endometriotic lesions do not necessarily regress during pregnancy; they can remain stable or even increase in size [36]. 

Limited data on reoperation rates are available in the literature. Regarding reoperation rates, our study reported an 8.0% rate during long-term follow-up. Choi et al. showed a five-year follow-up reoperation rate in women with OMA of 8.3% [37]. In comparison, Rindos et al. showed a higher reoperation rate in the long-term follow-up period (17.95%) [21]. 

Surgery is not only essential for the diagnostic process in women with suspected peritoneal endometriosis but is also one therapeutic cornerstone apart from conservative medication. Our study helps to counsel patients before the surgical excision of endometriotic lesions regarding the long-term outcome in terms of QoL impact and reoperation rate regarding the different subtypes of this disease. 

Addressing potential biases, we have to stress that our collective is not homogenous regarding confounding factors—although low in number—like ongoing pregnancy, breastfeeding, hormonal therapy, or patients who had undergone a hysterectomy due to adenomyosis. One limitation of our study is that it was conducted at a single center, which may influence the generalizability of the results.

One strength of our study is the postoperative long-term follow-up period of a median of 48 months. Moreover, our research stands out for its thorough sub-analysis regarding endometriosis subtypes and hormonal influence. 

We assume that further research studies with longer follow-up periods and larger study cohorts are necessary to draw definitive conclusions on the long-term QoL impact of surgical resection of endometriotic implants. These studies should focus on a holistic primary endpoint like the EHP-30, as it reflects all possible drawbacks caused by this enigmatic disease. 

## 5. Conclusions

The surgical removal of endometriotic lesions seems to be an effective long-term treatment option for patients with endometriosis who have an impaired QoL. Our study demonstrates that the surgical removal of endometriotic lesions offers significant improvements in the QoL of patients with endometriosis based on EHP-30 at a follow-up of 48 months. Positive effects on QoL after surgery were seen in patients with all endometriosis subtypes; however, women with DIE and adenomyosis had the strongest effect. Since only a few studies have evaluated the long-term impact on QoL after the surgical removal of endometriotic lesions based on the EHP-30, more studies are needed to confirm these results. 

## Figures and Tables

**Table 1:** Patient characteristics and clinical and pregnancy data at the long-term follow-up (*n* = 87 [100%]). IQR, interquartile range; releasing-IUS, releasing intrauterine system.

Variables	*n* = 87 (100%)
**age in years, (median [IQR])**	36 (31–42)
**age at menarche, (median [IQR])**	22.23 (20.7–25.3)
**MI kg/ m², (median [IQR])**	13 (12–14)
**smoker, n (%)**	20 (23.0)
**currently living in partnership, n (%)**	66 (75.9)
**follow-up in months, (median [IQR])** **reoperation, n (%)** yes no	48 (39–56) 7 (8.0%) 80 (92.0%)
**duration of bleeding in days, (median [IQR])**	5 (4–6)
**hormonal therapy at long-term follow-up, n (%)**	
yes	16 (18.4)
no	71 (81.6)
**type of hormonal therapy, n (%)**	
hormone releasing-IUS	9 (10.3)
combined oral hormonal contraceptives	4 (4.6)
oral progestin	2 (2.3)
contraceptive ring	1 (1.1)
**days with pain per month, n (%)**	
0	43 (49.4)
1–5	34 (39.1)
6–10	6 (6.9)
>10	4 (4.6)
**common endometriosis symptoms, n (%)**	
dysmenorrhea	43 (49.4)
dyspareunia	43 (49.4)
negative influence on sex life	20 (23)
dysuria	5 (5.7)
dyschezia	19 (21.8)
rectal bleeding	7 (8)
diarrhea	15 (17.2)
obstipation	21 (24.1)
**undergone hysterectomy, n (%)**	9 (10.3)
**desire to have children, n (%)**	
yes	33 (37.9)
no	54 (62.1)
**patients pregnant since surgery, n (%)**	28 (32.18)
**total pregnancies, n**	49
currently pregnant	5 (10.2)
vaginal delivery	17 (34.69)
cesarean section	14 (28.57)
ectopic pregnancy	2 (4.08)
abortion	11 (22.45)
**currently breastfeeding, n (%)**	8 (9.2)

**Table 2:** Endometriosis subtypes, rate of reoperation, and hormonal influence (hormonal treatment, pregnancy, or breastfeeding).

Entity	n (%)	Reoperation n (%)	Under Hormonal Influence n (%)	Undergone Hysterectomy n (%)
all patients	87 (100)	7 (8.0)	29 (33)	9 (10.3)
peritoneal	15 (17.2)	2 (13.3)	4 (26.7)	2 (22.2)
OMA	19 (21.8)	4 (21.1)	7 (36.8)	0 (0)
DIE	31 (35.6)	0 (0)	13 (41.9)	0 (0)
DIE + OMA	14 (16.1)	1 (7.1)	5 (35.7)	0 (0)
adenomyosis	8 (9.2)	0 (0)	0 (0)	7 (77.8)

**Table 3:** Multivariate analysis: impact of patient characteristics on EHP-30 results.

Predictor	Estimate	CI Low	CI High	*p* Value
age	−0.072	−0.148	0.000	0.064
smoking	−0.224	−1.203	0.737	0.653
hormone intake	−1.144	−2.357	0.017	0.068
pregnancies since surgery	−2.499	−4.546	−0.905	0.009

## Data Availability

The data used in this study are not publicly available due to privacy or ethical restrictions.

## Supplementary Materials

The following supporting information can be downloaded at: https://www.mdpi.com/article/10.3390/jcm13185641/s1, Endometriosis Health Profile-30 questionnaire.
