# Supplementary material for: Long-Term Follow-Up of the Quality of Life of Endometriosis Patients after Surgery: A Comparative Study
Source: J Clin Med. 2024 Sep 23;13(18):5641. doi: 10.3390/jcm13185641 (PMC11433490; doi:10.3390/jcm13185641)
Supplement: Supplementary file 1 [file jcm-13-05641-s001.zip › jcm-3209205-supplementary.pdf]

University of Oxford National Endometriosis Society

The Endometriosis Health Profile Questionnaire (EHP 30)

© Nuffield Department of Obstetrics & Gynaecology
& Health Services Research Unit
University of Oxford

In collaboration with The National Endometriosis Society

- *This questionnaire has been developed to measure the effect endometriosis has upon a woman's quality of life.*
- *Please answer all the questions.*
- *We are aware that you may have had endometriosis for a long time. We also understand that how you feel now may be different to how you have felt in the past. However, please would you answer the questions only in relation to the effect that endometriosis has had on your life* ***during the last 4 weeks.***
- *There are no right or wrong answers, so please tick the answers which best represent your feelings and experiences.*
- *Due to the personal nature of some of the questions please understand that you do not have to answer any questions if you would prefer not to.*
- *The information and answers you give will be treated with the utmost confidentiality.*
- *If you have any problems or would like any help or assistance with the completion of this questionnaire please contact Mrs Gill Spencer-Webb (01865 225208) who will be happy to help you.*
- *Once you have completed the questionnaire please could you return it in the pre-paid envelope provided.*
- *We would like to thank you very much in anticipation for taking the time to help us with this important research and we look forward to receiving your answers.*
- *This research is being funded with an educational grant from Pharmacia, USA.*

Part 1: Core Questionnaire

During the last 4 weeks, how often because of your endometriosis have you…..

	Never	Rarely	Sometimes	Often	Always
**1.** Been unable to go to social events because of the pain?	☐	☐	☐	☐	☐
**2.** Been unable to do jobs around the home because of the pain?	☐	☐	☐	☐	☐
**3.** Found it difficult to stand because of the pain?	☐	☐	☐	☐	☐
**4.** Found it difficult to sit because of the pain?	☐	☐	☐	☐	☐
**5.** Found it difficult to walk because of the pain?	☐	☐	☐	☐	☐
**6.** Found it difficult to exercise or do the leisure activities you would like to do because of the pain?	☐	☐	☐	☐	☐
**7.** Lost your appetite and/or been unable to eat because of the pain?	☐	☐	☐	☐	☐

Please check that you have ticked ***one box for each question*** before moving onto the next page

DURING THE LAST 4 WEEKS, HOW OFTEN BECAUSE OF YOUR ENDOMETRIOSIS HAVE YOU…..	Never	Rarely	Sometimes	Often	Always
**8.** Been unable to sleep properly because of the pain?	☐	☐	☐	☐	☐
**9.** Had to go to bed/lie down because of the pain?	☐	☐	☐	☐	☐
**10.** Been unable to do the things you want to do because of the pain?	☐	☐	☐	☐	☐
**11.** Felt unable to cope with the pain?	☐	☐	☐	☐	☐
**12.** Generally felt unwell?	☐	☐	☐	☐	☐
**13.** Felt frustrated because your symptoms are not getting better?	☐	☐	☐	☐	☐
**14.** Felt frustrated because you are not able to control your symptoms?	☐	☐	☐	☐	☐

Please check that you have ticked ***one box for each question***
before moving onto the next page

DURING THE LAST 4 WEEKS, HOW OFTEN BECAUSE OF YOUR ENDOMETRIOSIS HAVE YOU…..

	Never	Rarely	Sometimes	Often	Always
**15.** Felt unable to forget your symptoms?	☐	☐	☐	☐	☐
**16.** Felt as though your symptoms are ruling your life?	☐	☐	☐	☐	☐
**17.** Felt your symptoms are taking away your life?	☐	☐	☐	☐	☐
**18.** Felt depressed?	☐	☐	☐	☐	☐
**19.** Felt weepy/tearful?	☐	☐	☐	☐	☐
**20.** Felt miserable?	☐	☐	☐	☐	☐
**21.** Had mood swings?	☐	☐	☐	☐	☐
**22.** Felt bad tempered or short tempered?	☐	☐	☐	☐	☐

Please check that you have ticked ***one box for each question*** before moving onto the next page

DURING THE LAST 4 WEEKS, HOW OFTEN BECAUSE OF YOUR ENDOMETRIOSIS HAVE YOU…..	Never	Rarely	Sometimes	Often	Always
**23.** Felt violent or aggressive?	☐	☐	☐	☐	☐
**24.** Felt unable to tell people how you feel?	☐	☐	☐	☐	☐
**25.** Felt others do not understand what you are going through?	☐	☐	☐	☐	☐
**26.** Felt as though others think you are moaning?	☐	☐	☐	☐	☐
**27.** Felt alone?	☐	☐	☐	☐	☐
**28.** Felt frustrated as you cannot always wear the clothes you would choose?	☐	☐	☐	☐	☐
**29.** Felt your appearance has been affected?	☐	☐	☐	☐	☐
**30.** Lacked confidence?	☐	☐	☐	☐	☐

Please check that you have ticked ***one box for each question***
before moving onto Part 2

Part 2: Modular Questionnaire

Section A: These questions concern the effect endometriosis has had on your work **during the last 4 weeks**.

If you have not been in paid or voluntary employment during the last 4 weeks please tick here ☐ and move onto Section B.

DURING THE LAST 4 WEEKS, HOW OFTEN BECAUSE OF YOUR ENDOMETRIOSIS HAVE YOU…..

	Never	Rarely	Sometimes	Often	Always
**1.** Had to take time off work because of the pain?	☐	☐	☐	☐	☐
**2.** Been unable to carry out duties at work because of the pain?	☐	☐	☐	☐	☐
**3.** Felt embarrassed about symptoms at work?	☐	☐	☐	☐	☐
**4.** Felt guilty about taking time off work?	☐	☐	☐	☐	☐
**5.** Felt worried about not being able to do your job?	☐	☐	☐	☐	☐

Section B: These questions concern the effect endometriosis has had on your relationship with your child/children **during the last 4 weeks**.

If you do not have any children please tick here ☐ and move onto Section C.

DURING THE LAST 4 WEEKS, HOW OFTEN BECAUSE OF YOUR ENDOMETRIOSIS HAVE YOU…..

	Never	Rarely	Sometimes	Often	Always
**1.** Found it difficult to look after your child/children?	☐	☐	☐	☐	☐
**2.** Been unable to play with your child/children?	☐	☐	☐	☐	☐

Please check that you have ***answered each section*** before moving onto the next page

Section C: These questions concern the effect endometriosis has had on your sexual relationships **during the last 4 weeks.**

If you do not wish to answer, please tick here ☐ and move onto Section D.

HOW OFTEN DURING THE LAST 4 WEEKS BECAUSE OF YOUR ENDOMETRIOSIS HAVE YOU…..

	Never	Rarely	Sometimes	Often	Always
**1.** Experienced pain during or after intercourse? *If not relevant please tick here* ☐	☐	☐	☐	☐	☐
**2.** Felt worried about having intercourse because of the pain? *If not relevant please tick here* ☐	☐	☐	☐	☐	☐
**3.** Avoided intercourse because of the pain? *If not relevant please tick here* ☐	☐	☐	☐	☐	☐
**4.** Felt guilty about not wanting to have intercourse? *If not relevant please tick here* ☐	☐	☐	☐	☐	☐
**5.** Felt frustrated because you cannot enjoy intercourse? *If not relevant please tick here* ☐	☐	☐	☐	☐	☐

Please check that you have ***answered each section*** before moving onto the next page

Section D: These questions concern your feelings **during the last 4 weeks** about the medical profession.

If this section is not relevant to you please tick here ☐ and move onto Section E.

DURING THE LAST 4 WEEKS, HOW OFTEN BECAUSE OF YOUR ENDOMETRIOSIS HAVE YOU………

	Never	Rarely	Sometimes	Often	Always
**1.** Felt the doctor(s) you have seen is (are) not doing anything for you?	☐	☐	☐	☐	☐
**2.** Felt the doctor(s) think it is all in your mind?	☐	☐	☐	☐	☐
**3.** Felt frustrated at the doctor(s) lack of knowledge about endometriosis?	☐	☐	☐	☐	☐
**4.** Felt like you are wasting the doctor(s) time?	☐	☐	☐	☐	☐

Section E: These questions concern your feelings **during the last 4 weeks** about your treatment for endometriosis. Treatment means any surgery or **prescribed** medication for your endometriosis.

If this question is not relevant to you please tick here ☐ and move onto Section F.

DURING THE LAST 4 WEEKS HOW OFTEN BECAUSE OF YOUR ENDOMETRIOSIS HAVE YOU………

	Never	Rarely	Sometimes	Often	Always
**1.** Felt frustrated because treatment is not working?	☐	☐	☐	☐	☐
**2.** Found it difficult coping with the side effects of treatment?	☐	☐	☐	☐	☐
**3.** Felt annoyed at the amount of treatment you have had to have?	☐	☐	☐	☐	☐

Please check that you have ***answered each section***
before moving onto the next page

Section F: These questions concern your feelings **during the last 4 weeks** about any difficulties you might have conceiving.

If this section is not relevant to you please tick here ▢ and move onto Part 3.

DURING THE LAST 4 WEEKS, HOW OFTEN BECAUSE OF YOUR ENDOMETRIOSIS HAVE YOU………

	Never	Rarely	Sometimes	Often	Always
**1.** Felt worried about the possibility of not having children/more children?	▢	▢	▢	▢	▢
**2.** Felt inadequate because you may not/have not been able to have children/more children?	▢	▢	▢	▢	▢
**3.** Felt depressed at the possibility of not having children/more children?	▢	▢	▢	▢	▢
**4.** Felt that the possibility of not conceiving/not being able to conceive has put a strain upon your personal relationship?	▢	▢	▢	▢	▢

Please check that you have ***answered each section*** that applies to you before moving onto Part 3

Part 3: General Details

In this section, please could you tell us some general details about yourself………..

	Day	Month	Year
1. What is your date of birth?	☐☐	☐☐	☐☐☐☐
2. What date did you complete this questionnaire?	☐☐	☐☐	☐☐☐☐
3. When did you first have symptoms of endometriosis?			☐☐☐☐
4. When was your endometriosis first diagnosed at surgery?			☐☐☐☐

5. Please tick which symptom(s) you have had because of your endometriosis over the last 4 weeks *(you may tick more than one)*

a) Pelvic pain unrelated to period pain? ☐

b) Felt sick or vomited? ☐

c) Felt tired/lacking in energy? ☐

d) Pain when passing urine? ☐

e) Pain when opening bowels? ☐

f) Constipation or diarrhoea? ☐

g) Irregular bleeding? ☐

h) Period pain? ☐

Note: if you have not had a period during the last 4 weeks please tick here ☐

6. Are you *(please circle as appropriate)*

a) Single b) Cohabiting c) Married d) Separated e) Divorced f) Widowed

7. Please state where you filled in this questionnaire *(please circle as appropriate)*

a) at home b) in hospital c) other (please state)…………………………..

Please check that you have ***answered each section*** that applies to you before moving onto Part 4

Part 4: General Health Questions

---

1. Overall, how would you rate your health during the past 4 weeks?

Excellent	Very good	Good	Fair	Poor	Very Poor
☐	☐	☐	☐	☐	☐

---

2. During the past 4 weeks, how much did physical health problems limit your usual activities (such as walking or climbing stairs)?

Not at all	Very little	Somewhat	Quite a lot	Could not do physical activities
☐	☐	☐	☐	☐

---

3. During the past 4 weeks, how much difficulty did you have doing your daily work, both at home and away from home, because of your physical health?

None at all	A little bit	Some	Quite a lot	Could not do daily work
☐	☐	☐	☐	☐

---

4. How much bodily pain have you had during the past 4 weeks?

None	Very mild	Mild	Moderate	Severe	Very Severe
☐	☐	☐	☐	☐	☐

---

5. During the past 4 weeks, how much energy did you have?

Very much	Quite a lot	Some	A little	None
☐	☐	☐	☐	☐

Please check that you have ***answered each section***
before moving onto the next page

Part 4: continued

---

6. During the past 4 weeks, how much did your physical health or emotional problems limit your usual social activities with family or friends?

Not at all	Very little	Somewhat	Quite a lot	Could not do social activities
☐	☐	☐	☐	☐

---

7. During the past 4 weeks, how much have you been bothered by emotional problems (such as feeling anxious, depressed or irritable)?

Not at all	Slightly	Moderately	Quite a lot	Extremely
☐	☐	☐	☐	☐

---

8. During the past 4 weeks, how much did personal or emotional problems keep you from doing your usual work, school or other daily activities?

Not at all	Very little	Somewhat	Quite a lot	Could not do daily activities
☐	☐	☐	☐	☐

Now that you have completed the questionnaire please could you return it to us in the pre-paid envelope provided. Once again we would like to thank you for taking the time to help us with this research.

Note: Parts 1 and 2; EHP-30 and Modular Questions, © University of Oxford 2002

Part 4; SF-8™ 4-Week Recall Version - © 1999-2001 – QualityMetric, Inc. - All Rights Reserved
